# Mesoporous Materials Make Hydrogels More Powerful in Biomedicine

**DOI:** 10.3390/gels9030207

**Published:** 2023-03-09

**Authors:** Huangqin Chen, Xin Qiu, Tian Xia, Qing Li, Zhehan Wen, Bin Huang, Yuesheng Li

**Affiliations:** 1 Department of Stomatology, School of Stomatology and Ophthalmology, Xianning Medical College, Hubei University of Science and Technology, Xianning 437100, China; 2 Hubei Key Laboratory of Radiation Chemistry and Functional Materials, Non-Power Nuclear Technology Collaborative Innovation Center, Hubei University of Science and Technology, Xianning 437100, China

**Keywords:** mesoporous, hydrogel, biomedicine, application

## Abstract

Scientists have been attempting to improve the properties of mesoporous materials and expand their application since the 1990s, and the combination with hydrogels, macromolecular biological materials, is one of the research focuses currently. Uniform mesoporous structure, high specific surface area, good biocompatibility, and biodegradability make the combined use of mesoporous materials more suitable for the sustained release of loaded drugs than single hydrogels. As a joint result, they can achieve tumor targeting, tumor environment stimulation responsiveness, and multiple therapeutic platforms such as photothermal therapy and photodynamic therapy. Due to the photothermal conversion ability, mesoporous materials can significantly improve the antibacterial ability of hydrogels and offer a novel photocatalytic antibacterial mode. In bone repair systems, mesoporous materials remarkably strengthen the mineralization and mechanical properties of hydrogels, aside from being used as drug carriers to load and release various bioactivators to promote osteogenesis. In hemostasis, mesoporous materials greatly elevate the water absorption rate of hydrogels, enhance the mechanical strength of the blood clot, and dramatically shorten the bleeding time. As for wound healing and tissue regeneration, incorporating mesoporous materials can be promising for enhancing vessel formation and cell proliferation of hydrogels. In this paper, we introduce the classification and preparation methods of mesoporous material-loaded composite hydrogels and highlight the applications of composite hydrogels in drug delivery, tumor therapy, antibacterial treatment, osteogenesis, hemostasis, and wound healing. We also summarize the latest research progress and point out future research directions. After searching, no research reporting these contents was found.

## 1. Introduction

Porous materials are a kind of network structure material composed of interconnected or closed holes. Usually, porous materials have a huge surface area and connected pore structure, which can provide a channel for material transmission and diffusion, just like the transmission system of plant leaf veins in nature and the respiratory and blood circulation system of animals. According to the definition of the International Union of Pure and Applied Chemistry (IUPAC), porous materials can be simply classified based on their pore size. Materials with a pore size of less than 2 nm are microporous materials, including zeolites [1] and metal–organic frames (MOFs) [2], while those with a pore size larger than 50 nm are macroporous materials, with major representative materials such as aerogels [3] and porous ceramics [4]. When the pore size is between 2 nm and 50 nm, porous materials are named mesoporous materials.

The discovery of mesoporous materials revolutionized the field of porous materials from the study of zeolite to the later soft and hard template methods [5]. Mesoporous materials have been widely used in catalysis [6], biosensing [7], biomolecular adsorption and separation [8], drug delivery [9], regenerative medicine [10], and other fields due to their performance advantages, such as large surface area, high porosity, orderly and controllable pore size, and good hydrothermal stability.

A hydrogel is a kind of polymer with a three-dimensional network structure that can absorb water swelling and keep the original structure unchanged. It has good biocompatibility, excellent flexibility, structural designability, and functional adjustability [11]. In addition, hydrogels can sense small changes in external stimuli (such as temperature and pH value) and achieve their response to stimuli by swelling and contracting [12]. The excellent properties of hydrogels have aroused the interest of the majority of scientific research workers and made them widely used in several fields. In environmental pollution control, hydrogels can be used to adsorb heavy metal ions [13] and in dye wastewater treatment [14]. In biomedicine, bioactive substances can be simply mixed in hydrogel precursors and encapsulated in porous and expansive three-dimensional networks, which have interesting bionic properties and are widely used in tissue engineering [15], drug delivery [16], tumor therapy [17], and wound dressings [18]. Although hydrogels have many advantages, their poor mechanical properties, undesirable thermal stability, and drug burst release usually restrict their application [19]. In order to solve these problems, researchers have made many attempts in recent years. Examples include combining bioactive and functional nanomaterials with hydrophilic polymers to obtain enhanced physicochemical and biological properties [20]; to release drugs in a more stable, slower, and sustained manner, reducing sudden release [21]; and to endow hydrogels with more biological properties such as photothermal effects [22].

In this review, we first introduce the classification of composite hydrogels loaded with mesoporous materials, including silicon-based and non-silicon-based mesoporous material-loaded hydrogels, and then we briefly introduce the crosslinking methods of composite hydrogels loaded with mesoporous materials, namely physical crosslinking, chemical crosslinking, and radiation crosslinking. Subsequently, the latest progress in the use of composite hydrogels in different biomedical applications, such as drug delivery, tumor therapy, antibacterial, osteogenesis, and hemostasis, is summarized. Finally, the possible problems and future development directions of composite hydrogels are summarized in order to provide a reference for scientific research workers in related fields. After searching, no research reporting these contents was found.

## 2. Classification of Mesoporous Material-Loaded Composite Hydrogels

**Figure 1:**
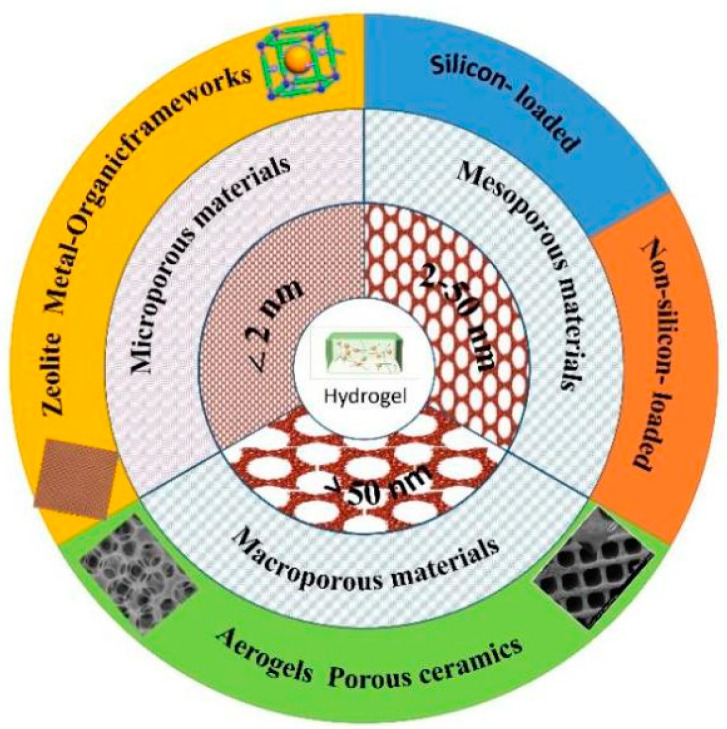
The classification of porous material-loaded composite hydrogels.

According to the chemical composition of the loaded mesoporous materials, the composite hydrogels can be divided into mesoporous silicon-based and non-silicon-based loaded hydrogels. Figure 1 shows the classification of porous material-loaded composite hydrogels. Table 1 shows the classification of mesoporous material-loaded composite hydrogels.

### 2.1. Mesoporous Silicon-Loaded Composite Hydrogels

#### 2.1.1. Mesoporous Silica-Loaded Composite Hydrogels

Mesoporous silica is an inorganic nonmetallic material with uniform and adjustable pore size, large surface area, functionalize modifiable surface, and good biocompatibility [67]. Since 1992, when Mobil Company developed the silicon-based mesoporous material M41S (MCM-41, MCM-48, MCM-50) series for the first time [68], researchers have developed the SBA series [69,70], MCF series [71], and MSU series [72] successively. Such versatile nanomaterials have undergone over two decades of evolution, and now, a number of hybrid analogs of mesoporous silica nanoparticles have also been fabricated based on the rapid development of nanosynthetic chemistry, such as the well-known periodic mesoporous organosilicas and mesoporous organosilica nanoparticles [73]. Researchers have been focused on the research and development of mesoporous silica composite hydrogels and have found that loading with mesoporous materials can effectively improve various properties of hydrogels and expand their application range. For example, the addition of mesoporous silica MCM-41 to poly (aspartic acid) hydrogels significantly enhanced their thermal stability, liquid absorption ratio, and salt tolerance [74]. Mixing SBA-15 with spruce xylan/2-hydroxyethyl methacrylate hydrogels improved their mechanical properties by increasing the surface area and maximizing the interaction between the inorganic particles and the polymer matrix, as well as preventing cracks and slowing the breakage of hydrogels [29]. Combining MCM-41 or SBA-15 as drug carriers into carboxymethyl cellulose hydrogels not only increased the in vitro swelling, erosion, water vapor/oxygen permeability, and antibacterial activity [26], but also enabled the loaded drugs to be completely released after 12 h and improved the in vitro mucoadhesion of the hydrogels [75]. Before being embedded in a hydrogel, modification with appropriate functional groups of mesoporous silica can obviously improve the application limitation of pure silicon-based mesoporous materials, such as the loading of hydrophobic drugs and the controlled release of loaded drugs. Phosphonate-functionalized mesoporous silica with amino and phosphonic ester groups increased the water solubility of the insoluble drug by 7-fold, and mesoporous silica-loaded gel showed a significantly higher amount of retention of the drug in the skin [76]. Carboxylated mesoporous silica loaded into a gelatin methacryloyl hydrogel not only increased the mechanical strength, compressive modulus, and swelling ratio of the hydrogel, but also led to a controlled release of metformin, endowing the hydrogel with better biocompatibility, antibacterial activity, and superior stem cell adhesion and proliferation induction [77]. The alginate/chitosan polyelectrolyte-modified mesoporous silica nanoparticles also improved the mechanical properties of aldehyde hyaluronic acid/N, O-carboxymethyl chitosan hydrogels and achieved sustained drug release due to the Schiff base reaction between the aldehyde group in aldehyde hyaluronic acid and the amino group in chitosan [78].

#### 2.1.2. Mesoporous Bioglass-Loaded Composite Hydrogels

Bioactive glass is a silicate glass composed of SiO_2_, Na_2_O, CaO, and P_2_O_5_. So far, bioactive glass is the only artificial biological material that can not only bond with bone tissue, but also connect with soft tissue, which can repair, replace, and regenerate body tissue. Therefore, the functional development of bioactive glass mainly focuses on bone tissue engineering. Mesoporous bioactive glasses have a highly ordered system of pores, a larger pore volume, and significantly increased specific surface area, making them more biologically active than pure silica mesoporous materials [79].

Incorporation with mesoporous bioactive glasses is considered a promising way to improve the physicochemical stability and mechanical properties of the hydrogels in hard tissue engineering [80,81,82]. At the same time, the combined mesoporous bioactive glass can optimize the drug delivery and release performance of hydrogels, improving the efficacy of loaded drugs [48,83]. A naringin or calcitonin gene-related peptide co-printed into the mesoporous bioactive glass/sodium alginate/gelatin hydrogels displayed a steady sustained release behavior for up to 21 days without an initial burst release and promoted MG-63 cell proliferation and osteogenesis-related gene expression [84]. Preserving the bioactivity of growth factors and realizing the two coupled processes of osteogenesis and angiogenesis are challenging in the development of bone regeneration. A composite hydrogel composed of mesoporous bioactive glass achieved differentiated delivery modes for osteogenic recombinant human bone morphogenetic protein-2 and angiogenic vascular endothelial growth factor [85] and promoted osteogenic differentiation of primary mouse bone marrow stromal stem cells and angiogenesis [86]. More than that, a mesoporous bioactive glass-loaded composite hydrogel improved bone regeneration in some extreme conditions, such as osteoporosis [87], oxidative stress [88], and tumor microenvironments [89].

### 2.2. Mesoporous Non-Silicon-Loaded Composite Hydrogels

Since the chemical inertness of silica restricted the application of silicon-based mesoporous materials, scientists started trying to synthesize non-silicon mesoporous materials, such as mesoporous metal oxides. Mesoporous titanium dioxide can serve as an effective photosensitizer and initiator for the formation of hydrogel and protect the loaded drugs from being degraded into an inactive form during multi-mode antitumor therapy [57]. Mesoporous zinc oxide also has the function of drug delivery [62]. The addition of mesoporous zinc oxide to the hydrogel successfully alleviates the undesired burst release of the loaded drug, resulting in the sustained release at a neutral pH over time [61]. Mesoporous manganese dioxide has unique properties of alleviating localized hypoxia by inducibly promoting the decomposition of H_2_O_2_ present in inflammatory settings [90]. A hydrogel encapsulated with mesoporous manganese dioxide can relieve localized oxidative stress and prolonged oxygen deprivation via effective oxygen generation [91]. Mesoporous manganese dioxide also can help composite hydrogels achieve satisfactory glutathione/pH/thermal-responsive controlled drug release in the tumor microenvironment, which is attributed to the fact that manganese dioxide is a redox-active material and can be degraded under glutathione, H_2_O_2_, and acid conditions [64,92]. Mesoporous polydopamine is a new material with good biocompatibility and excellent photothermal properties, which can effectively convert near-infrared light into heat and kill cancer cells in vivo and in vitro [93]. The abundant groups on the surface of polydopamine can be combined with drugs through chemical bonding, electrostatic adsorption, and π–π stacking to improve the drug load rate, prolong the drug release time, and reduce the burst release behavior [94]. In addition, the infrared absorption property of mesoporous polydopamine can be utilized to accelerate the drug release rate through near-infrared light irradiation, which makes mesoporous polydopamine/cellulose nanofibril hydrogels have potential applications in both chemical and physical therapy [95].

## 3. Crosslinking Methods of Mesoporous Material-Loaded Composite Hydrogels

The crosslinking methods of composite hydrogels can be categorized as physical crosslinking, chemical crosslinking, and radiation crosslinking. Table 2. Shows the classification of preparation methods.

### 3.1. Physical Crosslinking Method

Physical crosslinking is the crosslinking between linear molecules formed by hydrogen bonding, electrostatic interaction, coordination bonding, and hydrophobic interaction. Pineapple peel carboxymethyl cellulose/polyvinyl alcohol/mesoporous silica SBA-15 hydrogels were synthesized by an eco-friendly method of repeated freeze–thaw cycles. The results of Fourier transform infrared spectroscopy analysis indicated that there was a slight shift of the band corresponding to O-H stretching vibration occurred and meanwhile the shifted bands became stronger during the hydrogel preparation, without any chemical reaction [99]. Using sodium alginate-g-P (NIPAM90-co-NtBAM10) as a gelator, a mesoporous silica composite hydrogel was prepared with dual pH- and thermo-responsiveness. During preparation, the cationic polymer (bearing amino groups) and the anionic polymer (bearing carboxyl groups) consecutively covered the mesoporous silica layer by layer, making the incorporation of the P(NIPAM90-co-NtBAM10) side chains in the anionic polyelectrolyte layer strengthen the hydrophobic interactions responsible for the formation of the network crosslinks [104].

### 3.2. Chemical Crosslinking Method

The chemical crosslinking method is divided into the crosslinker method and the chemical reaction method. In the crosslinker method, a crosslinker is used is to make the polymer aqueous solution form a network structure. The use of an initiator (2, 2-Methylpropionitrile) and crosslinker (ethylene glycol dimethacrylate) for poly (HEMA-co-AA)@ZnO composites avoided degradation of the copolymer and improved the stability of the system [61].

In chemical reaction method, the functional groups in the molecular chain of a polymer react with each other to form covalent bonds; examples include the thiololefn click reaction [100], Schiff base reaction [78], free-radical polymerization [101], and hybridization chain reaction [31]. A gel formed by chemical crosslinking has higher mechanical properties and no melting or dissolving.

### 3.3. Radiation Crosslinking Method

Radiation crosslinking mainly uses light (visible light, ultraviolet light, near-infrared light, etc.), γ rays, and electron beams to produce free radicals and initiate crosslinking reactions so that linear molecules of main chains of polymers are connected by chemical bonds [105]. When compared to the above methods, the radiation crosslinking method has a lot of advantages, such as high efficiency, simple operation, room temperature reaction, and being non-polluting [106]. A mesoporous silica-COOH methacrylate gelatin hydrogel was prepared by photocrosslinking using a light-emitting diode light-curing unit for 30 s [77]. A mesoporous titania@methylene blue/polyethyleneglycol diacrylate hybrid hydrogel was prepared via photopolymerization using methylene blue-sensitized mesoporous titania nanocrystals as a photosensitizer and photoinitiator. Under laser irradiation, mesoporous titania@methylene blue composites are stimulated to generate photoelectrons, which can interact with polyethyleneglycol diacrylate for the generation of radicals and then initiate radical polymerization and hydrogel formation [57].

## 4. Application of Mesoporous Material-Loaded Composite Hydrogels

**Figure 2:**
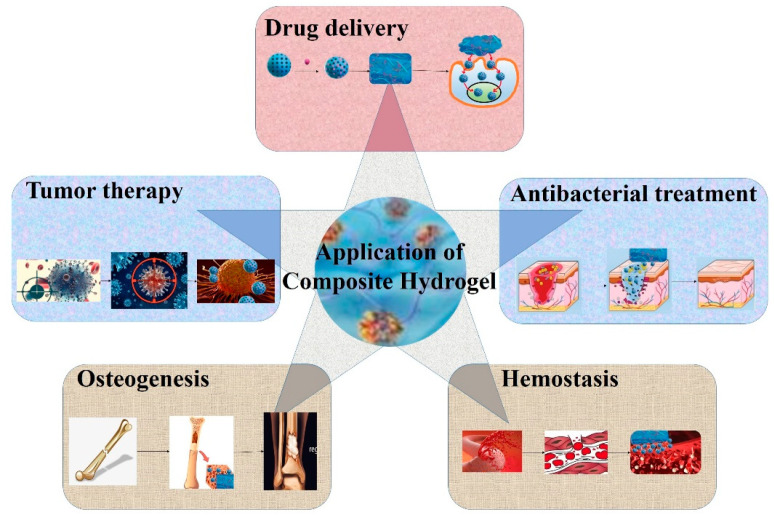
The application of mesoporous material-loaded composite hydrogels.

In recent years, hydrogels have been widely used in the field of biomedicine, especially in tissue regeneration and controlled drug release. However, further clinical application is limited by their shortcomings, such as weak adhesion of wet surfaces, low thermal stability, poor mechanical properties, and uncontrolled degradation [107]. The introduction of mesoporous materials, especially functionalized mesoporous materials, into hydrogels to synthesize composite hydrogels can not only improve the mechanical strength of hydrogels, but also make hydrogels have special functional properties and more extensive applications. Figure 2 displays the application of mesoporous material-loaded composite hydrogels.

### 4.1. Drug Delivery

**Figure 3:**
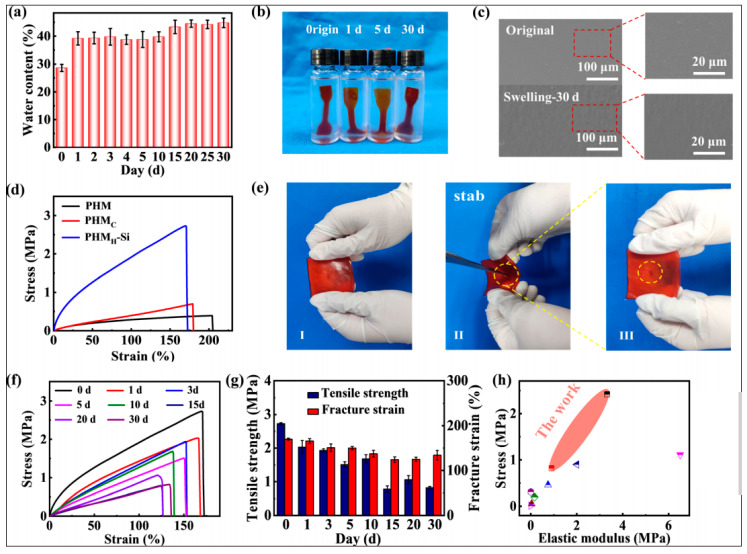
Swelling resistance and mechanical stability of the estradiol-loaded mesoporous silica- poly (hydroxyethyl methacrylate) (PHEMA) hydrogel (PHMH-Si). (**a**) The swelling rate of the PHMH-Si hydrogel in simulated uterine fluid for 30 days. (**b**) The actual pictures of the PHMH-Si hydrogel immersed in the simulated uterine cavity fluid for 0, 1, 5, and 30 days. (**c**) SEM of the PHMH-Si hydrogel before and after swelling in the simulated uterine fluid for 30 days. (**d**) Tensile stress–strain curves of the hydroxyl methacrylate/maleic anhydride (PHM), PHM/N, N-methylenebisacrylamide (PHMC), and PHMH-Si hydrogels. (**e**) Macroscopic photographs demonstrating the puncture resistance of the PHMH-Si hydrogel via scissors. (**f**) Stress–strain curves and (**g**) elastic moduli–fracture toughness histogram of the PHMH-Si hydrogel soaked in simulated uterine fluid for 30 days. (**h**) Mechanical properties of the PHMH-Si hydrogel in the as-prepared state (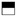) and after swelling in the simulated uterine fluid for 30 days (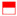), and other as-prepared PHEMA-based hydrogels [108].

With the development and progress of medicine, the design and construction of a new drug delivery system with high efficiency and low toxicity has become an urgent task. Encapsulation of drugs in a hydrogel precursor can no longer meet the increasing requirements of drug delivery. Porous mesoporous materials have unique advantages in carrying drugs, making up for the insufficiencies of hydrogels. In the early stage, the purpose of adding mesoporous materials to a hydrogel drug delivery system was to reduce the explosion effect and increase stability. Activated mesoporous silica in composite hydrogels was shown to be the determining factor in inhibiting nearly 90% of the initial burst of the drug, allowing the drug to be released with minimal burst kinetics [27]. The mesoporous silica also endowed the composite hydrogel with superior mechanical properties, more excellent stability, and good biocompatibility similar to that of the pure hydrogel, allowing it to maintain its structure and mechanical properties in simulated uterine fluid for 30 days. In addition, the hydrogel consistently and steadily released loaded drug, showing a good therapeutic effect in promoting endometrial cell proliferation and inhibiting fibrosis progression [108] (Figure 3).

**Figure 4:**
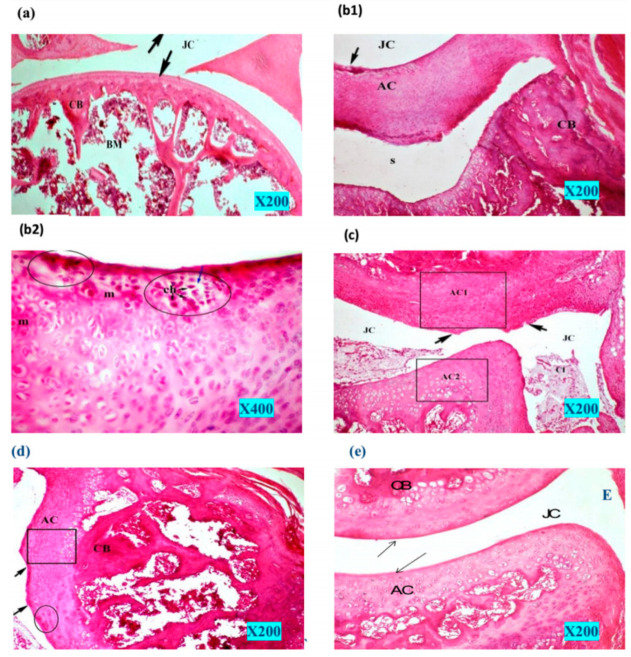
Histopathology photomicrographs of knee joint tissue. Normal control group (**a**), mono-iodoacetate (MIA) control group (**b1**), and higher magnification (**b2**). Free colchicine aqueous solution-treated rat group (**c**). Formula 1 (transdermal hydrogel patch comprising colchicine)-treated rat group (**d**). Formula 2 (the transdermal hydrogel patch comprising the equivalent amount of colchicine encapsulated in mesoporous silica nanoparticles)-treated rat group (**e**). Articular hyaline cartilage (AC), joint cavity (JC), compact bone (CB), bone marrow (BM), chondrocytes (Ch), cellular infiltration (CI) [110].

Later, mesoporous materials were used to help loaded drugs overcome the shortcomings of low water solubility and bioavailability, or high water solubility and poor skin permeability. The natural product curcumin is a promising compound in Alzheimer’s disease treatment. However, its water insolubility, low bioavailability, and instability in the biological environment hampered its clinical application. Loading curcumin onto mesoporous silica in a hydrogel exhibited excellent mechanical properties and much higher penetration values in pig nose mucosa than free curcumin [109]. Colchicine was observed as a potential treatment for osteoarthritis. Extensive first-pass effects, poor bioavailability, and serious gastrointestinal tract side effects in oral administration have led scientists to explore the transdermal route, which also has some limitations, such as high aqueous solubility and hence poor skin permeation. Using mesoporous silica as colchicine encapsulators revealed enhanced drug flux and amplified permeated drug levels in comparison to the free drug aqueous solution [110]. The nearly normal intact bone architecture reflects the potentiality of nanoencapsulation in mesoporous silica for enhancing drug delivery to the affected tissue (Figure 4).

Recently, with the advent of smart hydrogels, mesoporous materials used as drug reservoirs wrapped in smart hydrogels have been found to achieve improved load capacity and prolonged release time, even achieving stimulation-responsive release. Ordered mesoporous carbon embedded in injectable thermosensitive hydrogels progressively released its payload at a slower rate than the control gel (without ordered mesoporous carbon) [111]. Mesoporous silica in temperature and light dual-response mechanisms provided additional non-collapsible space for drug storage and demonstrated higher loading capacity and greater release ability [112]. pH/thermo-responsive grafted mesoporous silica controlled the release kinetics of a model drug through environmental conditions, accelerating the release of the drug with increasing pH and/or temperature under sink conditions [101]. More than that, mesoporous materials homogeneously distributed in hydrogels met the requirement of being a biocompatible carrier for the controlled delivery of bioactive compounds. The release of proteins, such as bone morphoprotein 2 and bovine serum albumin, from hydrogels tends to involve a burst release stage. However, loading them into mesoporous silica in the injectable hydrogels with a designed core/shell carrier follows zero-order kinetics [113].

Achieving synergistic therapeutic effects through the dual delivery of different bioactive molecules is another challenge in the development of drug delivery systems. Mesoporous organosilica as co-administration carriers of a hydrophobic drug and oxygen in nanocomposite hydrogels is proven to improve drug loading, sustained delivery, and cell viability under both normoxia and hypoxia conditions [114].

### 4.2. Tumor Therapy

**Figure 5:**
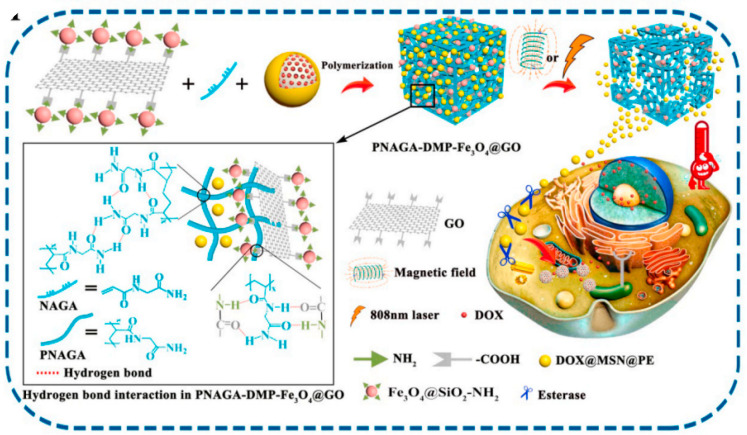
Composite hydrogels for tumor therapy [116].

Antitumor platinum drugs have several drawbacks, such as high toxicity and acquired tumor resistance, limiting their long-term and high-dose administration. Mesoporous silica nanoparticles impregnated with platinum drugs were developed to diminish toxicity and increase anticancer effectiveness, which also opened the door for the active targeting of nanoparticles through various functionalization [115]. Mesoporous material modification and surface functionalization through hydrogels can achieve tumor targeting or micro-environmental stimulation responsiveness with significantly increased drug concentration at tumor sites. This delivery system effectively changes the distribution of tumor drugs in vivo and reduces damage to normal cells. Hyaluronic acid-modified mesoporous silica composite hydrogels, self-assembling in situ around the tumor tissue by hydrogen bonds between hyaluronic acid molecules, have a higher affinity to tumor cells due to the overexpression of CD44 receptors on their surface. The prepared hydrogels gradually release the drug container (mesoporous silica), followed by endocytosis and intracellular drug release. In this process, anchored hyaluronic acid plays multiple roles as a tumor-targeting point and a drug release cap [98]. The mesoporous material with a magnetic core–shell structure can also be enriched in tumor tissue, effectively improving the targeting of the carrier under the action of an external or internal magnetic field. Ferrosoferric oxide nanoparticle-grafted graphene oxide endows controlled release of payload-loaded mesoporous silica under a magnetic field and near-infrared irradiation, resulting in the proper quantity of a therapeutic dose in tumor cells and negligible premature leakage of antitumor drugs to healthy tissue [116] (Figure 5).

Moreover, the introduction of responsive mesoporous material further accurately manipulates the sustained release of local drugs under various requirements of the tumor environment. Biocompatible mesoporous bioactive glass nanoparticles, pH-sensitive drug nanocarriers, and a tumor acidity neutralizer were designed as an in situ injectable hydrogel for topical and sustained drug (deoxyribonucnase I) release to neutralize tumor acidity and degrade neutrophil extracellular traps [117]. Mesoporous silica, responsible for the loading and local delivery of growth factors, can be localized in a tumor environment (37.5 °C, pH 6.8) by immediately switching from nanoparticles to compact hydrogels due to the pH-triggered hydrogen bonds. The increase in pH to 7.4 after the cure of the tumor initiated the cleavage of hydrogen bonds and the sustained release of growth factors, which promoted the proliferation of healthy cells [118]. In addition to tumor acidity, excess glutathione can also be used as a stimulator for smart responsive drug nanocarriers in the tumor-specific redox microenvironment. Since the disulfide bond in cysteamine is glutathione-sensitive, hydrophilic cytarabine encapsulated into the mesoporous silica with the hydrogel of sodium hyaluronate/cysteamine achieved successively controlled delivery at a high concentration of glutathione [119]. Based on the fact that the surfactant trimethoxyoctadecylsilane can present different on/off states in different solvents, an intelligent “on/off” switching strategy of site-specific co-loading of MnOx and Ce6 into mesoporous silica was proposed for the first time. The multifunctional theranostic nanoplatform shows a sensitive longitudinal relaxation signal in response to the overexpressed hydrogen peroxide and weak acidic pH in the tumor microenvironment [120].

**Figure 6:**
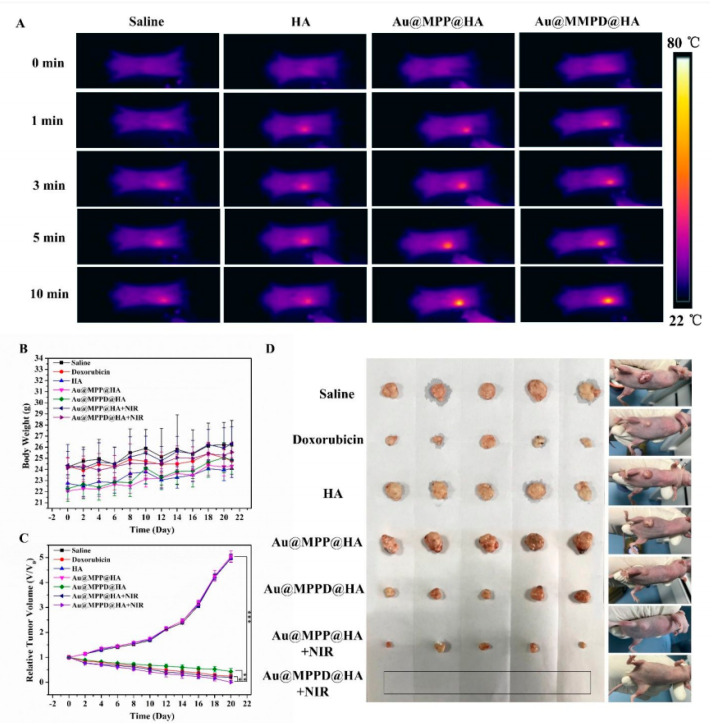
In vivo antitumor activities of nanocomposite hydrogels. (**A**) Thermographic photos of mice irradiated with near-infrared irradiation (NIR) laser irradiation at 0, 1, 3, 5, and 10 min (808 nm, 1 W/cm^2^). (**B**) Body weight changes of experimental mice. (**C**) Relative tumor weight changes of mice. (**D**) Representative images of harvested tumors from corresponding mice after different treatments [121].

In recent years, the emergence of nanotechnology has provided more strategies for tumor treatment, such as photothermal therapy and photodynamic therapy initiated by light. Core–shell gold mesoporous silica nanoparticles (Au@MPP) conjugated with triphenylphosphine and hyaluronic acid hydrogel (Au@MPPD@HA) not only selectively attack cancer cells and then enter into mitochondria, but also mediate the transformation of near-infrared radiation into thermal energy, enhancing cellular injury [121] (Figure 6). Indocyanine green-loaded mesoporous silica packaged together with a bio-oxygen pump (cyanobacteria Synechococcus UTEX 2973) was used to establish an injectable hydrogel that can support stable oxygen production and transfer energy to generate cytotoxic reactive oxygen species to promote the therapeutic effect [122].

### 4.3. Antibacterial Treatment

**Figure 7:**
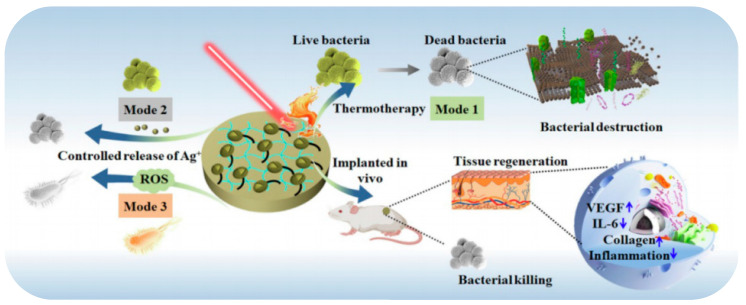
Composite hydrogel for antibacterial treatment [130].

The introduction of antibacterial drugs [97,123,124,125], antimicrobial peptides [126], or other antibacterial substances into the three-dimensional network structure of hydrogels through mesoporous materials is one of the most proven and effective methods to enhance the antibacterial and anti-inflammatory effect of hydrogels. Mesoporous materials loaded with antibacterial substances display overwhelming anti-inflammatory and antibacterial effectiveness with longer time and smaller doses [127]. Among them, hydrogels with Ag, Au, Cu, and other transition metal ions, oxides, or photocatalytic antibacterial agents modified by mesoporous materials have tremendous therapeutic potential and broad application space. The core–shell nanostructure of biomimetic virus-like mesoporous silica-coated Ag nanocubes entrapped in a hydrogel dressing is more capable of effectively adsorbing on the rigid cell wall of both *Escherichia coli* and *Staphylococcus aureus* than antibacterial silver nanoparticles [128]. The incorporation of mesoporous silica-modified CuS nanoparticles [129] or Ag_2_S quantum dots [130] (Figure 7) into the network structure of hydrogels was used to build a multi-modal therapeutic platform with the combined effects of hyperthermia, radical oxygen species, and metal ions released under near-infrared irradiation against *Escherichia coli* and methicillin-resistant *Staphylococcus aureus*.

**Figure 8:**
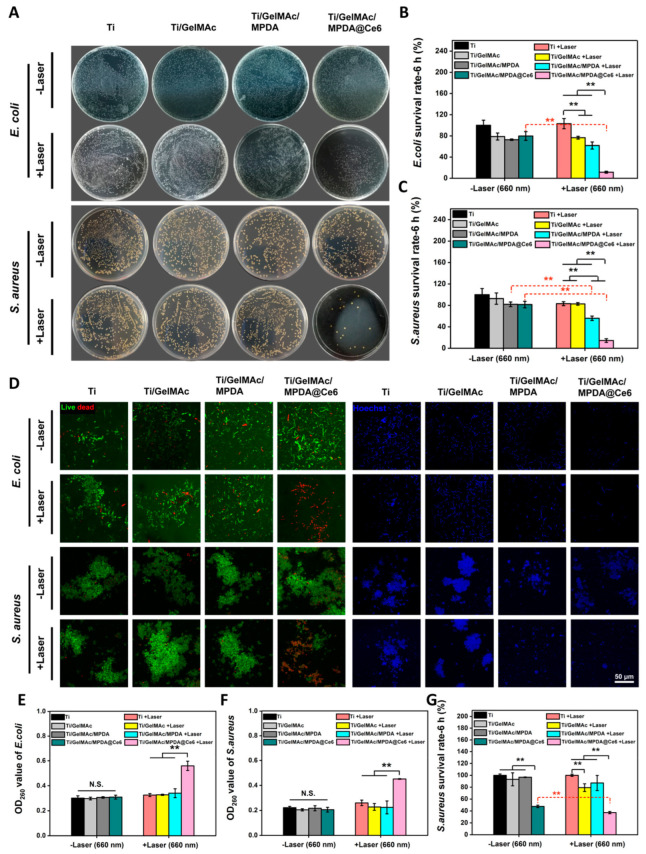
In vitro antibacterial tests. In these tests, *S. aureus* and *E. coli* were incubated with different samples upon laser irradiation (1 W/cm^2^) and cultured for 24 h. (**A**) Bacterial colony plate counting photos. (**B**,**C**) The corresponding quantitative analysis according to colony plate counting. (**D**) Representative images of live/dead staining and adhesion tests. (**E**,**F**) OD260 values of *S. aureus* and *E. coli* after incubation with different samples. (**G**) MTT assay measuring the viability of *S. aureus* after laser irradiation and further 6 h of incubation (100 mW/cm^2^) (n = 6, ** *p* < 0.01) [131].

Mesoporous polydopamine itself is a near-infrared light absorption material, and its photothermal conversion efficiency is much higher than that of some other common photothermal materials, such as metal-based nanoparticles, carbon-based nanomaterials, and organic polymers. Photodynamic therapy can also be enhanced by loading photosensitizers into mesoporous polydopamine as into other photothermal nanomaterials. Loading photosensitizer Chlorin e6 into mesoporous polydopamine by π–π stacking in a hydrogel system protects the efficacy of photosensitizer and achieves tight adhesion, improved stability, and remarkable antibacterial activity of the coating owing to the encapsulated mesoporous polydopamine [131] (Figure 8). Loading N,N’-disecbutyl-N,N’-dinitroso-p-phenylenediamine, a UV-vis light-responsive nitric oxide donor, into mesoporous polydopamine of a fibrinin-based hydrogel displays the near-infrared-triggered release of nitric oxide against methicillin-resistant *Staphylococcus aureus* infection with the help of the photoconductivity, strong light-harvesting, and electron transport abilities of mesoporous polydopamine [66].

### 4.4. Osteogenesis

**Figure 9:**
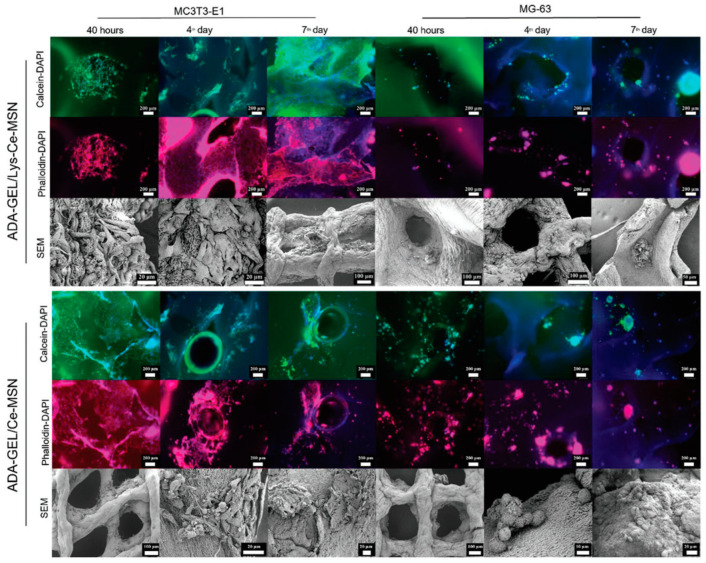
Morphology of MC3T3-E1 and MG63 cells adhered on scaffolds imaged by fluorescence microscopy (after Calcein/DAPI and Phalloidin/DAPI staining) and SEM after 40 h, 4 days, and 7 days of incubation [56].

Hydrogels have become a research hotspot in bone repair systems due to their porous three-dimensional structure and flexible flow pattern which allow the gradual release of loaded substances and minimally invasive administration. However, the low mineralization degree and poor mechanical properties of hydrogels after implantation mean that hydrogels have difficulty in providing the required mechanical support, which severely restricts their application in weight-bearing bone defect restoration [132]. Combination with inorganic nanoparticles, especially mesoporous materials, is one of the effective strategies. As expected, mesoporous cerium-doped mesoporous silica-calcia nanoparticles raised the mechanical stiffness of the composite hydrogel, induced the surface apatite mineralization, and promoted proliferation, adhesion, and differentiation of preosteoblast cells [56] (Figure 9).

**Figure 10:**
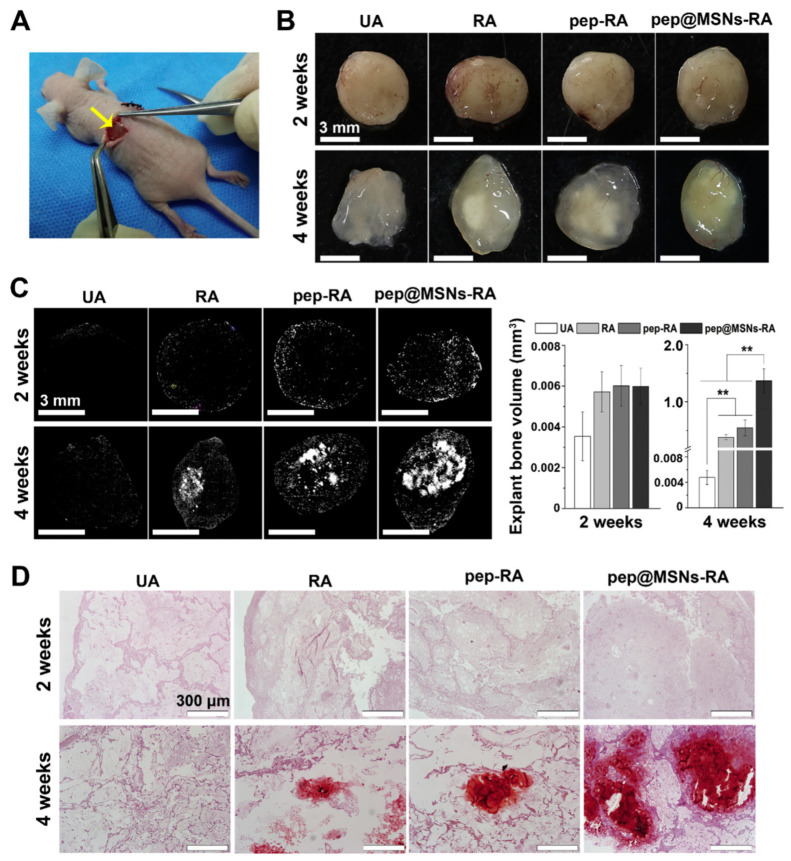
Visualized gross view and quantified volume of mineralized matrix in different human mesenchymal stem cell (hMSC)-loaded gels in vivo. (**A**) Gels encapsulated with hMSCs were subcutaneously implanted into nude mice. (**B**) Gross view of different hMSC-loaded gels following removal from mice 2 and 4 weeks post-surgery. (**C**) Micro-CT reconstruction images of gel mineralization and quantitative bone volume analysis after 2 and 4 weeks of implantation. (**D**) Alizarin Red-S (ARS) staining of the implanted hMSC-loaded gels after 2 and 4 weeks of subcutaneous implantation. The *p* values were calculated by Tukey’s post hoc test (** *p* < 0.01). All data represent mean ± SD (n = 3) [135].

Furthermore, the addition of 1 wt% mesoporous bioactive glass significantly enhanced the mechanical strength, matched the degradation rate and ionic dissolution of composite hydrogels, and exhibited excellent biocompatibility and osteoinductivity in vitro while remarkably promoting bone formation and inhibiting osteoclastogenesis in vivo [133]. Mesoporous materials can also be used as drug carriers in composite hydrogels to load and release various bioactivators to promote osteogenesis. Common examples include bone morphogenetic protein 2 [103], parathyroid hormone [134], and bone-forming peptide-1 [135] (Figure 10).

### 4.5. Hemostasis

**Figure 11:**
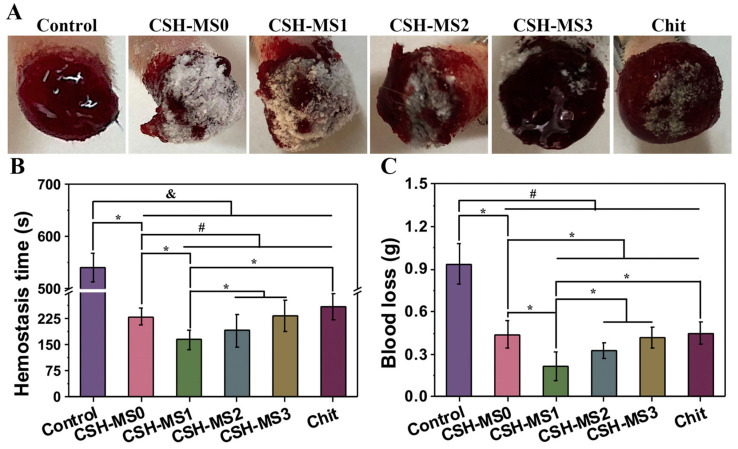
Hemostatic activity of cationic superabsorbent hydrogel coordinated by mesoporous silica (CSH-MS). (**A**) In vivo hemostatic performance. (**B**) Hemostasis time in different groups of materials applied to rat tail truncation model. (**C**) Blood loss in rat tail truncation model. * *p* < 0.05; # *p* < 0.01; & *p* < 0.001 [101].

Mesoporous materials have a large specific surface area and pore volume, which can quickly absorb a large amount of water in the blood and promote platelet coagulation. When a mesoporous material is used as a carrier of hemostatic drugs, it can also exert a hemostatic effect by hitting the bleeding site effectively and accurately. The introduction of functional groups to the mesoporous materials adjusts the surface properties (hydrophilic/hydrophobic) and pore size and, more importantly, improves the biocompatibility and coagulation function. Chitosan- and hydrocaffeic acid-modified mesoporous silica nanoparticles, which enhanced the mechanical strength of the blood clot and activated the coagulation cascade, showed shorter hemostatic time than that of mesoporous silica in the femoral artery trauma model of Sprague Dawley rats [136]. Hydrogels have the characteristic of adhesion besides keeping the wound moist, which enables the wound to be sealed quickly and thus accelerates hemostasis. A composite hydrogel coordinated by mesoporous silica greatly elevated the water absorption rate to about 5000%, dramatically shortened bleeding time, and reduced blood loss in a rat tail amputation model by plasma protein activation [101] (Figure 11).

### 4.6. Wound Healing and Tissue Regeneration

Wound healing is a successive process requiring multiple biological pathways and intricate biochemical cascade reactions to be activated and synchronized to recover tissue integrity [137]. Hydrogels are an ideal dressing for the wound management market in protecting the wound against bacterial infection, but impaired vessel formation and undermined cell proliferation make chronic wounds stall in the inflammatory phase [138]. Thus, developing hydrogels with capacities to promote tissue regeneration is highly desired. Incorporating mesoporous materials can be promising for enhancing the biological properties and antimicrobial activity of hydrogels. After integration, the composite hydrogels have excellent wound closure effects, boosted reactive oxygen species-scavenging activity, and significant angiogenesis ability [139]. They also can obtain better organized epidermal and dermal layers in full-thickness skin wounds in in vivo wound healing studies [140] and realize the integration of angiogenesis and infection inhibition [50].

Diabetic wounds characterized by impaired healing due to uncontrolled and interrelated bacterial infection, reduced vascularization, and hypoxia microenvironment remain a challenge worldwide. Co-encapsulation of mesoporous microspheres and antimicrobial substances in hydrogels is an effective combination to obtain complementary or cooperative biological functions. Carrying platelet-derived extracellular vesicles and resveratrol-loaded mesoporous silica nanoparticles in hydrogels reduced inflammation and oxidative stress in the wound microenvironment and promoted tube formation by human umbilical vein endothelial cells [141]. Co-encapsulation with metformin-loaded mesoporous silica microspheres and silver nanoparticles promoted fibroblast migration and endothelial cell angiogenesis in vivo via spatiotemporal immunomodulation [142]. The encapsulation of ultrasmall Ag nanoclusters and deferoxamine-loaded polydopamine/hollow mesoporous manganese dioxide nanoparticles in hydrogels effectively alleviated localized oxidative stress and facilitated the healing of infected wounds through the sequential engagement of antibacterial, anti-inflammatory, and proangiogenic activities [91].

Recently, copper ions have emerged as promising agents in wound healing of diabetic wounds due to the stimulation effects on the proliferation and angiogenesis of cells. Glucose oxidase hybrid copper oxide nanoparticles in hydrogels produced H_2_O_2_ for antibacterial usage and induced the proliferation and migration of keratinocytes and tubule formation of endothelial cells by the simultaneously released Cu^2+^ [143]. N-isopropylacrylamide and acrylamide hydrogels controlled the release rate of copper ions during near-infrared light irradiation leading to both antibacterial effects and skin tissue regeneration [129]. Hydrogels containing photosensitizer Chlorin e6-loaded mesoporous polydopamine nanoparticles exhibited remarkable and rapid antibacterial activity through laser-triggered reactive oxygen species generation. After bacterial elimination, daily lower-power (100 mW/cm^2^) laser irradiation brought an excellent photobiomodulation effect through fibroblast activation [131].

Aphthous stomatitis, also known as canker sores, is a common oral cavity disease manifested by the presence of multiple painful yellow-grayish ovoid ulcers on the oral mucosa. A variety of new carriers, such as niosome, liposome-containing hydrogel, and solid lipid nanoparticle-containing hydrogel have been used in the treatment, but there have been no reports on mesoporous material-loaded hydrogels [144].

## 5. Patents and Clinical Trails

In the past five years, a variety of patents on the improvement of preparation methods of mesoporous material-loaded composite hydrogels have emerged. In terms of drug delivery, the incorporation of one or more drugs into the matrix of a hydrogel formed in situ to allow local drug release has been realized [145]. A slow-release carrier can even be prepared with only natural material without violent reaction [146]. The preparation of antitumor composite hydrogels is still focused on the combined application of photothermal photosensitive therapy and chemotherapy [147]. As for wound healing, composite hydrogels with antibacterial, adhesive, hemostasis, self-healing, and other functions are the focus of research, and mesoporous materials mainly involve silica and polydopamine [148,149]. Bone defect repair is another important application direction for composite hydrogels. Patents related to various stimuli-responsive composite hydrogels [150,151], composite hydrogels that can regulate local reactive oxygen species [152], and three-dimensional bioprinting [153] have been reported. However, there are few reports on clinical trials of composite hydrogels loaded with mesoporous materials.

## 6. Conclusions and Prospect

The synthesis of mesoporous materials began in the 1990s, and since then, scientists have been continuously exploring performance improvement and application expansion. Both silicon-based and non-silicon-based mesoporous materials can be combined with hydrogels to play a synergistic role in biomedicine through physical crosslinking, chemical crosslinking, and radiation crosslinking. Uniform mesoporous structure, high specific surface area, excellent adsorption, and especially the pore size of mesoporous materials which matches the size of proteins, nucleic acids, and various compounds make them suitable for the adsorption, transportation, and sustained release of multiple active substances in hydrogels. Modification and surface functionalization of mesoporous materials by hydrogels ensures a drug delivery system with tumor site targeting and tumor microenvironment responsiveness and provides more strategies for tumor treatment, such as photothermal therapy and photodynamic therapy initiated by near-infrared radiation. In addition to introducing antibacterial drugs, antimicrobial peptides, or other antibacterial substances into hydrogels, mesoporous materials can also modify Ag, Au, Cu, and other transition metal ions, oxides, or photocatalytic antibacterial materials, and even mesoporous materials themselves have photothermal conversion ability, which significantly improves the antibacterial ability of hydrogels and offers a new antibacterial mode. In bone repair systems, especially in weight-bearing bone defect restoration, mesoporous materials remarkably strengthen the mineralization and mechanical properties of hydrogels, aside from being used drug carriers to load and release various bioactivators to promote osteogenesis. Examples include bone morphogenetic protein 2, parathyroid hormone, and bone-forming peptide-1. Mesoporous materials have a large specific surface area and pore volume, which can greatly elevate the water absorption rate of hydrogels, enhance the mechanical strength of the blood clot, and dramatically shorten bleeding time.

Although the research on mesoporous material-loaded composite hydrogels is still progressing, there are still some problems worth paying attention to: (1) From the perspective of the preparation method of composite materials, radiation preparation has the advantages of green quality and high efficiency, but there are few research reports on this method. (2) From the perspective of the types of composite materials, mesoporous silica and mesoporous bioactive glass are mainly focused on, while mesoporous carbon, mesoporous titanium dioxide, and mesoporous polydopamine have excellent photocatalytic properties and are expected to be used in photodynamic therapy. Therefore, future research can focus on these aspects to further expand the application of mesoporous material composite hydrogels.

## Figures and Tables

**Table 1:** Classification of mesoporous material-loaded composite hydrogels.

Mesoporous Material	Chemical Composition		Hydrogel	Properties	Reference
Silicon	Silica	MCM41	Chitosan/Alginate	pH-sensitive and sustained release	[23]
		MCM-41	Poly(N-isopropylacrylamide)	Improved mechanical property	[24]
		MCM-41	Poly[(2-acryloyloxy)ethyl trimethylammonium chloride]	High water absorbency	[25]
		MCM41,ZnO	Carboxymethyl cellulose	Great improvement in tensile strength, swelling, erosion, and gas permeability	[26]
	Silica	SBA-15, SBA-16	Alginate	Enhanced drug delivery	[27]
		SBA-15	Poly[(N-isopropylacrylamide)-co-(mathacrylic acid)])(P[(N-iPAAm)-co-(MAA)])	Thermo- and pH-sensitive	[28]
		SBA-15	Spruce xylan, 2-hydroxyethyl methacrylate	A potential scaffold for fibroblast growth and attachment	[29]
		KIT-6	Poly(N-isopropylacrylamide)	Thermo-responsive, controlled, and sustainable releases	[30]
	Silica		Acrylamide	Target stimulus, magnetic	[31]
			N-isopropylacrylamide, 2,2-dimethylaminoethyl methacrylate	Stimuli-responsive (pH, magnetic)	[32]
			Polyvinyl alcohol	Inferior stability	[33]
			Poly(N-isopropylacrylamide)	Thermo-responsive	[34]
			N,N-dimethacrylamide	Temperature response	[35]
			Polyamidoamines	Promoting mesenchymal stem cell migration	[36]
			Polyacrylamide polydopamine	Enhance strength and adhesiveness to skin	[37]
			Chitosan	pH- and electro-responsive	[38]
			Gelatin methacryloyl	Improve stem cell adhesion	[39]
			PEG-PCL-PEG	Near-infrared irradiation-controlled drug delivery	[40]
			Silk fibroin, oxidized sodium carboxymethyl cellulose	pH- and redox-responsive bi-drug administration	[41]
			Methacrylate gelatin /methacrylate hyaluronic acid	Sustained-release drug carrier	[42]
			Chitosan	Sustained-delivery capability	[43]
			Poly(ethylene glycol)-b-poly(lactic-co-glycolic acid)-b-poly(N-isopropylacrylamide)	Injectable thermo-responsive	[44]
			chitosan	pH-responsive, magnetic	[45]
	Organosilica		Alginate	Control cell growth and cell migration	[46]
			Alginate	Control the enrichment of cells and simultaneous drug delivery	[47]
	Bioglasses		Poly(ethylene oxide)-poly(propylene oxide)-poly(ethylene oxide)	Injectable thermosensitive	[48]
			Biopolymer	Injectable strong adhesion and removability reusability	[49]
			Modified hyaluronic acid	Injectable, long-term release	[50]
			Chitosan-gelatin	Osteoinductivity capacity	[51]
			Methylcellulose/polyvinyl Alcohol/polyvinylpyrrolidone	Injectable, thermo-sensitive	[52]
			Pluronic F-127	Thermosensitive behavior and injectability	[53]
			Methacrylate gelatin	Better mechanical property, durable degradation time, pH stable, biomineralization, and long-term ion release	[54]
	Silica-Calcia		Alginate dialdehyde-gelatin	Enhance osteoblast proliferation, adhesion, and differentiation	[55]
			Alginate dialdehyde-gelatin	Induce bone regeneration, address infection	[56]
Non-silicon	Metal Oxides	TiO_2_	Polyethyleneglycol diacrylate	Peritumoral injectability, localized and sustainable release, photochemistry effects	[57]
			Poly(ethylene glycol)	High biocompatibility	[58]
			Poly(ethylene glycol)	Yielded higher fluorescence signals and was more sensitive with lower detection	[59]
			Alginate salt	Excellent photocatalytic functions	[60]
		ZnO	Poly(2-hydroxyethyl methacrylate), poly(2-hydroxyethyl methacrylate)-co-poly(acrylic acid)	Mitigate undesirable burst-release effects	[61]
			PCL-PEG-PCL	Drug carriers	[62]
		MnO_2_	Fenugreek gum, methylenebisacrylamide), acrylamide	Selective and effective adsorption of organic dyes	[63]
			Poly(N-isopropylacrylamide-co-acrylic acid)	Thermal/pH-sensitive	[64]
	Polydopamine		Cellulose nanofibrils	pH and near-infrared responsiveness	[65]
			Gel	Injectable and biodegradable	[66]

**Table 2:** Classification of preparation methods.

Classification	Mesoporous Material	Hydrogel	Preparation Method	References
Physical crosslinking	Silica	Poly(d,l-lactide)-poly(ethylene glycol)-poly(d,l-lactide)	Simple mixture	[96]
	Polydopamine	Cellulose nanofibril	Hydrogen bonding and π−π stacking interaction	[95]
	Silica	Polyacrylate	Ionic bonding and multiple hydrogen bonds	[97]
	Silica	Hyaluronic acid	Self-assemble hydrogen bonds	[98]
	Silica	Pineapple peel carboxymethyl cellulose, polyvinyl alcohol	Freeze–thaw	[99]
Chemical crosslinking	Silica	3-((2-(methacryloyloxy) ethyl) dimethylammonio) propane-1-sulfonate	Thiololefn click reaction	[100]
	Silica	Aldehyde hyaluronic acid, N,O-carboxymethyl chitosan	Schiff base reaction	[78]
	Silica	Poly(ethylene glycol) diacrylate, [2-(Methacryloyloxy)ethyl] trimethylammonium chloride	Free-radical polymerization	[101]
	Silica	polyacrylamide	Hybridization chain reaction	[31]
	ZnO	Poly(2-hydroxyethyl methacrylate) Poly(HEMA-co-AA)	Initiator crosslinking method	[61]
	Silica	Methacrylate gelatin, methacrylate hyaluronic acid	Photo-initiated crosslinking method	[42]
Radiation crosslinking	Silica	α-cyclodextrin, hyaluronic acid	Self-assemble (near-infrared radiation)	[102]
	Bioglass	Methacrylate gelatin	Photo-crosslinked	[103]
	Titania	Polyethyleneglycol diacrylate	Photopolymerization under near-infrared radiation	[57]
